# No Association between Metabolic Syndrome and Periodontitis in Korean Postmenopausal Women

**DOI:** 10.3390/ijerph182111110

**Published:** 2021-10-22

**Authors:** Jeong-In Kim, Choong-Ho Choi, Ki-Ho Chung

**Affiliations:** 1 Department of Preventive & Public Health Dentistry, Chonnam National University, Gwangju 61186, Korea; kji6298@naver.com (J.-I.K.); hochoi@chonnam.ac.kr (C.-H.C.); 2 Dental Science Research Institute, School of Dentistry, Chonnam National University, Gwangju 61186, Korea

**Keywords:** postmenopausal, metabolic syndrome, periodontitis, oral health behavior

## Abstract

This study aimed to determine the association between metabolic syndrome (MetS) and periodontitis in Korean postmenopausal women. The study selected 3320 menopause women (40–79-year-old) from those who participated in the seventh Korea National Health and Nutrition Survey (2016–2018). This association was determined using frequency and multiple logistic regression analyses. The prevalence of MetS in postmenopausal women was 33.2%, and among the MetS components, abdominal obesity showed a higher odds ratio of periodontitis by 1.36 (95% confidence interval (CI): 1.12–1.65; *p* < 0.05). The odds ratio for periodontitis was 1.34 times higher (95% CI: 1.12–1.60) in the MetS prevalence group with three or more MetS components (*p* < 0.05); however, after adjusting for demographic characteristics and health behavior variables, it was not statistically significant. Therefore, our results indicated that MetS has no association with periodontitis in postmenopausal women after adjusting for confounding variables.

## 1. Introduction

According to Organization for Economic Cooperation and Development (OECD) Health Statistics 2019, the life expectancy of Korean women is 85.7 years, which is 2.3 years longer than the OECD average of 83.4 years [1]. As the average lifespan of women has increased, the span of middle and old age have been extended, interest in the health of postmenopausal women has increased, and importance of health management is evolving. The average age of the onset of menopause for women is 49.4 years [2], which can be a major turning point in terms of life and health for middle- and old-aged women.

Menopause is a state in which the ovarian function decreases and causes a decline in estrogen levels secreted by the ovaries and cessation of menstruation [3]. In women, menopause is known to increase the risk of metabolic syndrome (MetS) by 60% independent of age, body mass index, and physical activity beyond the simple cessation of menstruation [4]. Changes in the distribution of fat in the body, such as postmenopausal abdominal obesity, neutrophil counts, and total cholesterol levels increase the incidence of cardiovascular disease [5,6,7].

Owing to changes in diet and lifestyle, more than one disease exists simultaneously in modern society, including diabetes, hypertension, obesity, and hyperlipidemia [8]. MetS is a condition in which hypertension, diabetes, hyperlipidemia, and cardiovascular disease risk factors such as insulin resistance, advanced age, increased body mass index, and smoking are seen [6]. It has recently become clear that non-alcoholic fatty liver disease is a systemic disease and may play a key role in MetS [9]. MetS has also been reported to be associated with periodontal disease and tooth loss [10].

Periodontal disease is one of the most common chronic inflammatory diseases, and severe periodontitis causes periodontal tissue destruction, leading to tooth loss [11]. Periodontal disease is also known to induce or sustain a systemic chronic inflammatory state, facilitating arteriosclerosis and increasing the incidence of cardiovascular disease [12].

Various studies have reported the connection between metabolic diseases (such as obesity, cardiovascular disease, and diabetes) and periodontitis [13,14,15,16], and the connection between MetS and periodontitis is being elucidated [17,18,19,20,21]. Some studies have demonstrated the effect of adult MetS components on periodontitis, and this relationship has also been reported in Korea [16,21,22,23,24]; however, regional and global studies are insufficient for the relationship between menopause MetS and periodontitis.

Therefore, the hypothesis of this study was established that the MetS component of postmenopausal women was related to periodontal disease.

## 2. Materials and Methods

### 2.1. Study Population

The study was conducted in accordance with the Declaration of Helsinki and the protocol was approved by the Ethics Committee of the Institutional Review Board of Korea Centers for Disease Control and Prevention (2018-01-03-P-A).

The total number of participants who enrolled in the seventh Korea National Health and Nutrition Examination Survey (KNHANES; 2016–2018) was 24,269, and 3320 postmenopausal women (40–79-year-old) were included in this study, from the 16,489 individuals who participated in the oral examination.

### 2.2. Assessment of MetS

MetS was assessed on the following five components: (1) abdominal obesity (waist circumference of ≥ 85 cm for females); (2) high blood pressure (systolic ≥ 130 mmHg and diastolic ≥ 85 mmHg); (3) fasting serum glucose level ≥ 100 mg/dL; (4) hypertriglyceridemia (serum triglyceride > 150 mg/dL); (5) low high-density lipoprotein (HDL) cholesterol levels (<50 mg/dL for females).

MetS was diagnosed if three or more of the five components were identified using the National Cholesterol Education Program Adult Treatment Panel standard III [25] and abdominal obesity was assessed using the Korean Society for the Study of Obesity standard [26].

### 2.3. Assessment of Periodontitis

Periodontitis was assessed based on the Community Periodontal Index of Treatment Needs certified by the World Health Organization, in 1982, and uses the Community periodontal index (CPI) [27], which generally reflects periodontal conditions. The CPI score is classified as “0–2” no periodontitis and “3–4” periodontitis.

### 2.4. Assessment of Confounders

Among the variables surveyed in the seventh KNHANES (2016–2018), age, education, household income, smoking, drinking, stress, oral examination, daily toothbrushing frequency, use of oral hygiene products, and chewing and speaking challenges were used as variables. Ages were classified into “40–49 years,” “50–59 years,” “60–69 years,” and “70–79 years.” Educational level was classified as “≤elementary school,” “middle school,” “high school,” and “≥college;” household income level was classified as “Lowest,” “Second lowest,” “Second highest,” and “Highest” based on quartiles (households). Drinking was classified as “No” for not drinking at all in the past year, “Low” for less than once a month to less than four times a month, and “High” for more than 2–4 times a week. Smoking and occasional smoking were classified as “Current.” Consequently, those who smoked in the past but not currently smoked were classified as “Past” and those who never smoked were classified as “No.” Stress was classified as “Very severe,” “Severe”, “Occasionally,” and “Rarely.” Oral examinations were classified as “Yes” and “No” if they had or had not undergone oral examinations in the past 1 year, respectively. The daily brushing frequency was classified as “≤1,” “2,” and “≥3,” and the use of oral hygiene products was classified as “Yes” and “No.” Chewing and speech problems were classified as “uncomfortable” for very uncomfortable and uncomfortable and “comfortable” for mediocre, not uncomfortable, or not at all uncomfortable.

### 2.5. Statistical Analyses

A complex sample design was applied for the data analysis, and the statistical significance level of 0.05 was used for all analyses. Frequency analysis was performed to identify the participants’ socio-demographic characteristics, health status and behavior, MetS, and periodontitis. Multiple logistic regression analysis was performed to verify the difference in periodontitis according to MetS. The Statistical Package for the Social Sciences (SPSS), version 26.0 (IBM SPSS Statistics for Windows, Armonk, NY, USA) was used for all statistical analyses.

## 3. Results

### 3.1. MetS Characteristics

As a result of the analysis, abdominal obesity, hypertension, hyperglycemia, hypertriglyceridemia, and low-HDL cholesterol levels were seen in 35.9%, 38.6%, 43.1%, 27.7%, and 44.7% of the participants. The prevalence of MetS with three or more of these components was 33.2% (Table 1).

### 3.2. Periodontitis Prevalence according to Socio-Demographic Characteristics and Health Status and Behavior

As a result of analyzing the distribution of periodontal disease according to the socio-demographic characteristics and health status and behavior, the prevalence of periodontitis was the highest among those in their 70 s at 39.9% and the lowest at 25.7% in their 40 s. The prevalence of periodontal disease as per the education status was the highest at 42.6% in elementary school and below and the lowest at 28.8% in college and above. The prevalence of periodontitis as per the income level was the highest at 41.1% in the lowest group and the lowest at 31.4% in the highest group (*p* < 0.05, Table 2).

As a result of analyzing the distribution of periodontitis according to health status and behavior, the prevalence of periodontitis was the highest in current smokers at 57.7%. Moreover, 40.2% of individuals did not undergo oral examination in the last year, which was higher than those who underwent oral examination. The frequency of daily brushing was more than once in 41.1% of individuals, and proxabrush, dental floss, and mouthwash were not used by 38.0%, 39.4%, and 39.4% of individuals, respectively. Chewing caused discomfort in 44.3% of individuals, whereas there was comfortable in 33.7% of individuals (*p* < 0.05, Table 2).

### 3.3. Association between MetS and Its Components and Periodontitis

Logistic regression analysis was performed to verify the association between the MetS components and periodontitis. As a result of the analysis, the odds ratio was 1.36 times higher in the abdominal obesity group (*p* < 0.05), and it was not statistically significant in hypertension, hyperglycemia, hypertriglyceridemia, and low-HDL cholesterol groups (Table 3).

Multiple logistic regression analysis was performed to verify the association between MetS and periodontitis. As a result of the analysis, the odds ratio was 1.34 times higher in the MetS group (*p* < 0.05); however, it was not statistically significant after adjusting for socio-demographic characteristics and health status and behavior (*p* > 0.05, Table 3).

## 4. Discussion

In women, the prevalence of cardiovascular disease increases because of changes in female hormones related to menopause from the age of 40, which escalates the body fat accumulation, and physical changes with aging [28], and it is known that the cardiovascular disease mortality rate is high in menopausal women with MetS [29]. Additionally, as periodontal disease is closely related to systemic disease, it occurs or worsens because of weakened immunity and systemic diseases, such as respiratory and cardiovascular diseases, the prevalence of which increases with age [30]. It has been suggested that the common cause between periodontitis and MetS is inflammation [31]. Periodontal-produced inflammatory cytokines or periodontal pathogens/LPS enter the systemic circulation and thus affect other parts of the body, promoting or inducing the development of MetS [32]. It has been reported that a 10% increase in the number of sites with BOP is associated with increased diagnosis of MetS [33].

As a result of this study, the prevalence of MetS among postmenopausal women was determined as 33.2%, which was higher than the prevalence of MetS among the Korean adult women in 2008 (20.5%) and 2017 (18.7%) [34]. Lim et al. [35] showed a high prevalence of MetS in men under the age of 50 years and women above the age of 50 years, and menopause is known to increase MetS risk independent of age or body mass index and physical activity [4].

The periodontitis prevalence showed a high rate in the case of not receiving regular check-ups in the past year and less frequent brushing in health status and behavior [36,37], similar to the findings reported by Lee [38], who reported that the number of remaining teeth was high in the group that brushed more than three times daily. The prevalence of periodontal disease was higher in the group not using proxabrush, dental floss, or mouthwash, consistent with the study conducted by Han et al. [23], in which the risk of periodontal disease was 2.69 times higher in the non-flossing group of postmenopausal women, and the study of Park [39], where the prevalence of periodontal disease was high when proxabrush and dental floss were not used.

Among the MetS components, the prevalence of periodontitis was 1.36 and 1.34 times higher in the abdominal obesity and MetS prevalence groups, respectively; however, it was not significant after adjustment. This was significantly related only to the abdominal obesity group among MetS risk factors and was consistent with the results of the study conducted by LaMonte et al. [40], which showed that the prevalence of periodontitis was 1.74 times higher in the presence of supragingival plaque. According to the study results of Habashneh et al. [41], postmenopausal women with a BMI of 25 or higher had a lower risk of periodontitis compared to normal weight but showed a significantly higher loss of clinical attachment loss, plaque index, and calculus index, which partially coincided. Additionally, it was significant in diabetes and abdominal obesity, and it was consistent with the study results of Han [42], which showed a significant association with CPI as the number of risk factors for MetS increased. According to Kang et al. [43], the prevalence of periodontitis was significantly higher in fasting glucose, HDL cholesterol, and abdominal obesity, and the odds ratio for hypertension was significantly higher in the model corrected for health behavior and oral health behavior. According to a Kim [37], the risk of periodontitis in the hypertensive group was 1.60 times higher whereas the risk of periodontal disease was 2.22 times higher when 1 or more MetS risk factors were present. Additionally, it was partially consistent with the study result of Baek et al. [44], which showed that the risk of having periodontitis together was also high when three or more were present. Morita et al. [19] also found a 2.40 times higher risk of periodontitis in patients with MetS, Campos et al. [45] showed a 2.02 times higher prevalence of periodontitis in MetS patients, and a study by Gomes-Filho et al. [46] showed approximately 2 times higher probability of having moderate or severe periodontitis in individuals without periodontitis.

Similar to the findings [47], which suggest that cardiovascular disease risk factors are among the important causes of MetS in this study, it was established that menopause is an independent risk factor for periodontitis as abdominal obesity increases because of changes in body fat distribution. Thus, it is recommended that measures to prevent abdominal obesity in postmenopausal women should be explored.

This was a cross-sectional study that utilized the data from KNHANES to determine the association between MetS and periodontitis in Korean postmenopausal women, with a limitation of being unable to present an accurate causal association. MetS may only be a predictor, not a causal factor with periodontal disease. In addition, the use of hormone therapy in postmenopausal women and the drug use status of patients with metabolic diseases could not be investigated, which may have acted as a confounding variable in this study. However, using KNHANES data which possibly represent the national population, it was demonstrated that MetS is not associated with periodontitis in postmenopausal women. Thus, more in-depth studies are needed to determine the factors affecting the association between MetS and periodontitis in postmenopausal women.

## 5. Conclusions

We demonstrated that MetS has no association with periodontitis in postmenopausal women after adjusting for confounding variables.

## Figures and Tables

**Table 1:** Distribution of MetS and its components.

Variables	Classification	*n*	%
Abdominal obesity	<85 cm	2092	64.1
	≥85 cm	1219	35.9
Hypertension	<130/85 mmHg	1985	31.4
	≥130/85 mmHg	1333	38.6
Hyperglycemia	<100 mg/dL	1803	56.9
	≥100 mg/dL	138	43.1
Hypertriglyceridemia	<150 mg/dL	2295	72.3
	≥150 mg/dL	897	27.7
Low HDL cholesterol	≥50 mg/dL	1728	55.3
	<50 mg/dL	1461	44.7
MetS ^1^	Less than 3	2086	66.8
	3 or more	1094	33.2

Frequency analysis; ^1^ MetS is defined when three or more of abdominal obesity, hypertension, hyperglycemia, hypertriglyceridemia, low HDL-Cholesterol.

**Table 2:** Periodontitis according to socio-demographics characteristics and health status and behavior.

Variables	*n*	Periodontitis Status		*p*-Value ^1^
		Yes	No	
Age				
40–49	97 (100)	21 (25.7)	76 (74.3)	0.020
50–59	1106 (100)	376 (34.1)	730 (65.9)	
60–69	1170 (100)	464 (38.6)	706 (61.4)	
70–79	947 (100)	381 (39.9)	566 (60.1)	
Education				
≤Elementary school	1474 (100)	635 (42.6)	839 (57.4)	0.000
Middle school	571 (100)	199 (35.9)	372 (64.1)	
High school	830 (100)	279 (33.0)	551 (67.0)	
≥College	444 (100)	129 (28.8)	315 (71.2)	
Household income				
Lowest	1017 (100)	420 (41.1)	597 (58.9)	0.004
Second lowest	912 (100)	359 (38.4)	553 (61.6)	
Second highest	719 (100)	250 (35.0)	469 (65.0)	
Highest	663 (100)	209 (31.4)	454 (68.6)	
Drinking				
No	754 (100)	269 (35.0)	485 (65.0)	0.089
Low	1478 (100)	542 (35.8)	936 (64.2)	
High	229 (100)	104 (44.0)	125 (56.0)	
Smoking				
No	3095 (100)	1137 (36.2)	1958 (63.8)	0.000
Past	104 (100)	36 (32.7)	68 (67.3)	
Current	121 (100)	69 (57.7)	52 (42.3)	
Stress				
Very severe	176 (100)	69 (36.1)	107 (639)	0.342
Severe	647 (100)	264 (39.2)	383 (60.8)	
Occasionally	1771 (100)	628 (35.5)	1143 (64.5)	
Rarely	715 (100)	279 (39.3)	436 (60.7)	
Oral exam within 1 year				
No	2237 (100)	901 (40.2)	1336 (59.8)	0.000
Yes	1083 (100)	341 (30.3)	742 (69.7)	
Daily toothbrushing frequency				
≤1	315 (100)	122 (41.1)	193 (58.9)	0.008
2	1412 (100)	571 (39.6)	841 (60.4)	
≥3	1593 (100)	549 (33.9)	1044 (66.1)	
The use of proxabrush				
Non-use	2848 (100)	1087 (38.0)	1761 (62.0)	0.005
Use	472 (100)	155 (30.6)	317 (69.4)	
The use of dental floss				
Non-use	2749 (100)	1096 (39.4)	1653 (60.6)	0.000
Use	571 (100)	146 (25.6)	425 (74.4)	
The use of mouthwash				
Non-use	2379 (100)	944 (39.4)	1435 (60.6)	0.000
Use	941 (100)	298 (31.3)	643 (68.7)	
Chewing difficulty				
Discomfort	1100 (100)	483 (44.3)	617 (55.7)	0.000
No discomfort	2207 (100)	755 (33.7)	1452 (66.3)	
Speaking difficulty				
Uncomfortable	424 (100)	173 (41.4)	251 (58.6)	0.104
Comfortable	2883 (100)	1065 (36.4)	1818 (63.6)	

^1^ *p* < 0.05 using chi-square test.

**Table 3:** The association between MetS and periodontitis.

Variables	Model 1	Model 2
	Crude OR (95% CI ^†^)	AOR ^1^ (95% CI ^†^)
Abdominal obesity (ref. = No)	1.36 (1.12–1.65)	
Hypertension (ref. = No)	1.04 (0.87–1.23)	
Hyperglycemia (ref. = No)	1.11 (0.94–1.31)	
Hypertriglyceridemia (ref. = No)	1.08 (0.89–1.32)	
Low HDL cholesterol (ref. = No)	1.06 (0.89–1.27)	
MetS (ref. = No)	1.34 (1.12–1.60)	1.14 (0.92–1.42)

^1^ Adjusted odds ratio; ^†^ 95% confidence interval. Odds ratios and 95% confidence intervals are estimated using complex samples multivariable logistic regression analysis. Model 2 is an adjusted odds ratio considering age, education, household income, drinking, smoking, an oral examination within the past 1 year, daily toothbrushing frequency, the use of proxabrush, the use of dental floss, the use of mouthwash, and chewing and speaking difficulty.

## Data Availability

The dataset analyzed for this study can be found at https://knhanes.kdca.go.kr/knhanes/eng/index.do (accessed on 17 October 2021).
